# Highly dynamic inflammatory and excitability transcriptional profiles in hippocampal CA1 following status epilepticus

**DOI:** 10.1038/s41598-023-49310-y

**Published:** 2023-12-14

**Authors:** Daniel S. Galvis-Montes, Karen M. J. van Loo, Ashley J. van Waardenberg, Rainer Surges, Susanne Schoch, Albert J. Becker, Julika Pitsch

**Affiliations:** 1 Department of Epileptology, Medical Faculty, University of Bonn, Venusberg-Campus 1, 53127 Bonn, Germany; 2 Department of Epileptology, Neurology, RWTH Aachen University, Aachen, Germany; 3 i-Synapse, Cairns, Australia; 4 Section for Translational Epilepsy Research, Department of Neuropathology, University Hospital Bonn, Bonn, Germany

**Keywords:** Neuroimmunology, Transcriptomics

## Abstract

Transient brain insults including status epilepticus (SE) can initiate a process termed ‘epileptogenesis’ that results in chronic temporal lobe epilepsy. As a consequence, the entire tri-synaptic circuit of the hippocampus is fundamentally impaired. A key role in epileptogenesis has been attributed to the CA1 region as the last relay station in the hippocampal circuit and as site of aberrant plasticity, e.g. mediated by acquired channelopathies. The transcriptional profiles of the distinct hippocampal neurons are highly dynamic during epileptogenesis. Here, we aimed to elucidate the early SE-elicited mRNA signature changes and the respective upstream regulatory cascades in CA1. RNA sequencing of CA1 was performed in the mouse pilocarpine-induced SE model at multiple time points ranging from 6 to 72 h after the initial insult. Bioinformatics was used to decipher altered gene expression, signalling cascades and their corresponding cell type profiles. Robust transcriptomic changes were detected at 6 h after SE and at subsequent time points during early epileptogenesis. Major differentially expressed mRNAs encoded primarily immediate early and excitability-related gene products, as well as genes encoding immune signalling factors. Binding sites for the transcription factors *Nfkb1*, *Spi1, Irf8,* and two *Runx* family members, were enriched within promoters of differentially expressed genes related to major inflammatory processes, whereas the transcriptional repressors *Suz12, Nfe2l2* and *Rest* were associated with hyperexcitability and GABA / glutamate receptor activity. CA1 quickly responds to SE by inducing transcription of genes linked to inflammation and excitation stress. Transcription factors mediating this transcriptomic switch represent targets for new highly selected, cell type and time window-specific anti-epileptogenic strategies.

## Introduction

Epileptogenesis is the process by which changes occur in the brain after a precipitating injury or insult that leads to the development of epilepsy^1^. It involves the transition of a stable brain network into a functionally altered structure that leads to the development of spontaneously recurring seizures^2^. Temporal lobe epilepsy (TLE) is the most common form of focal epilepsy, which is often pharmacoresistant to antiseizure medication. Recent data suggest that SE induces highly variable gene expression in different cell types and compartments of the hippocampus^3–5^.

In experimental TLE models, including those triggered by kainic acid and pilocarpine-induced status epilepticus (SE), the hippocampal CA1 region appears to be an important site of epileptogenesis^6–8^. CA1 represents a key anatomo-functional compartment of the tri-synaptic pathway under epileptogenic conditions^9^. Only a few days after SE, the hippocampal CA1 subfield acquires the properties of an epileptogenic focus^10^.

Currently, it is believed that SE as a transient insult induces a specific transcriptional program that differs between distinct hippocampal subregions. Hereby, CA1 is of particular importance as it constitutes the output point of the hippocampal tri-synaptic circuit. Here, we aimed to elucidate the transcriptional cascades triggered shortly after SE and the key transcription factors (TFs) mediating these changes. So far, the dynamic transcriptional signatures at distinct pathogenic stages early after SE in the CA region have not been examined. Here, we provide for the first time a comprehensive analysis of the transcriptomic profiles of the early stages of epileptogenesis using bulk RNA-seq in hippocampal CA1 at 6, 12, 24, 36 and 72 h after pilocarpine-induced SE. Our results show these mRNA signatures to be highly dynamic during this period.

## Methods

### Animals

All animal procedures were planned and performed to minimize pain and suffering, and to reduce the number of animals used in accordance with European, national, and institutional guidelines (guidelines of the European Parliament and of the Council on the protection of animals used for scientific purposes, European Directive (2010/63/EU), and the ARRIVE guidelines). The study protocol was approved by the Landesamt für Natur, Umwelt und Verbraucherschutz (LANUV) of the state of North Rhine Westphalia, Germany; Ref. 8.87–50.10.31.08.119). All mice were housed in a humidity (55 ± 10%) and temperature (22 ± 2 °C) controlled environment under a 12-h light–dark cycle (light cycle 7 am to 7 pm) with water and food ad libitum, and nesting material (Nestlets, Ancare, USA). Mice were allowed to adapt to the facility for at least for seven days prior to any treatment.

### Induction of SE by systemic pilocarpine injection

For SE induction, adult (≥ 60 days old, weight ≥ 20 g) C57Bl/6N mice (Charles River) were pretreated with a subcutaneous injection of 1 mg/kg scopolamine methyl nitrate (Sigma), followed 20 min later by a subcutaneous injection of 335 mg/kg pilocarpine hydrochloride (Sigma), as previously described^11–13^. Mice were continuously visually observed after pilocarpine administration. Seizures were assessed behaviourally by the use of a modified seizure scheme, as described previously^11–13^. Sustained continuous convulsions with loss of postural were classified as SE as soon as they exceeded the threshold of 5 minutes^11,13^. Forty minutes after the onset of SE, animals received an injection of diazepam (4 mg/kg, s.c.; Ratiopharm). A single dose of diazepam has been shown to rapidly reduce clinical seizure activity and to lower mortality^11,13–15^. Control (non-SE) animals were treated identically, but received a saline injection instead of pilocarpine. Of the animals injected with pilocarpine, only those that developed SE (SE-experienced) were used for subsequent analyses.

### RNA isolation and library preparation

Mice were decapitated under deep isoflurane anesthesia (Forene, Abbott GmbH, Germany), brains were quickly removed and sectioned into 400 μm slices using a vibratome (Leica). Whole CA1 was carefully microdissected using two dissection needles by visual inspection in an optical microscope (Olympus). Total RNA was isolated using the miRNeasy micro kit (Qiagen) according to the manufacturer’s protocol. The quality and integrity of total RNA was assessed using a Tapestation 2200 (Agilent Technologies). RNA was considered as intact with an RNA Integrity Number (RINe) > 7. Purification of mRNA was performed using polyT oligo-attached magnetic beads. After purification, mRNA was used for library preparation using the TruSeq RNA Library Prep Kit v2 (Illumina) according to the manufacturer’s protocol. Sequencing was carried out on a HiSeq1500 system (Illumina) using cycle paired-end sequencing.

### Bioinformatic analysis

#### Mapping of RNA sequencing reads

The reference assembly for the Mus musculus genome (primary assembly; release GRCm39; soft masked) was obtained from Ensembl^16^. Gencode mouse annotations (release M26; gtf format) were used throughout^17^. For building genome indices and mapping, STAR version 2.7.8a was used. For genome indices, gtf and genome fasta files were provided to STAR as parameters –sjdbGTFfile and –geneFastaFiles with –runMode genomeGenerate to generate indices. –sjdbOverhand was set to 99 (read length—1)^18^. All other parameters remained default. Adapter trimming was performed using trimgalore version 0.6.6 (implemented in python version 3.8.5) and run in paired-end mode (using the –paired parameter)^19^. Adapters trimmed were “–illumina”. Sequence end soft clipping was performed using the default phred score of 20 and paired reads less than 20 bp for both reads were discarded. All other parameters were default. For mapping of RNA reads, STAR was run with parameter “–runMode alignReads” and paired (trimmed) reads input to the –readFilesIn parameter. Inputs for “—sjdbGTFfile” and “—genomeDir” parameters were the index described and gtf file described above. Non-default parameters were: –outSAMtype BAM Unsorted; –outFilterMultimapNmax 20; –outFilterMismatchNmax 999; –outFilterMismatchNoverReadLmax 0.04; –outFilterType BySJout; –alignIntronMin 20; –alignIntronMax 1000000; –alignMatesGapMax 1000000; –alignSJoverhangMin 8; –alignSJDBoverhangMin 1; –sjdbScore 1. Output bam files were sorted with samtools version 1.10and reads summarized using htseq-count version 0.13.5 (implemented with python version 3.8.5)^20,21^. Non-default htseq-count parameters included: –format bam; –order = pos; –stranded no. All other parameters remained default. After an initial assessment of lane or pair specific biases, reads from multiple lanes were merged into a single fastq files for each pair.

#### Differential expression analysis

For summarizing read counts, consensusDE version 1.8.0 was used and differential expression analysis using R version 4.05^22,23^. ConsensusDE simultaneously conducts statistical analysis using voom (limma version 3.46.0), edgeR (version 3.32.1), and DESeq2 (version 1.30.1) with the additional option to remove unwanted sources of variation with RUV, allowing comparison of the performance of these three algorithms with and without the application of RUV^24–27^. A summarized object of the read counts and annotations was created using the consensusDE “buildSummarized” function. Non-default parameters included a ‘sample_table’ describing the experimental design, directory of the htseq-count files (described above), path to the gtf file (described above) and “filter = TRUE” for filtering low-counts. Differential expression was then performed using the “multi_de_pairs” functions using the summarized object output from “buildSummarized” and the following non-default parameters: paired = "unpaired"; ruv_correct = TRUE, norm_method = "all_defaults". The gtf file described above was also used as the input to “gtf_annotate”. All other parameters remained default.

#### Gene ontology and Kyoto encyclopedia of genes and genomes (KEGG) enrichment analysis

Kyoto Encyclopedia of genes and genomes (KEGG)^28–30^ (pathway enrichment was performed using The Database for Annotation Visualization and Integrated Discovery (DAVID v 6.8). KEGG pathway analysis was used to determine the pathways consisting of differentially expressed genes (DEGs). Pathways without any link to DEGs were excluded from the analysis. To interpret the molecular function and biological process of the DEGs, Gene Ontology (GO) enrichment analysis was performed with the GO enrichment analysis and visualization tool (GOrilla), using the process, function and component ontology settings. TF-binding enrichment was performed, prioritizing TFs based on the overlap between DEG and TF targets assembled from ENCODE, ReMap, and individual publications; co-expression of TFs with other genes based on processed RNA-seq from TRANSFAC and JASPAR databases^31^. FDR corrected *p* values of 0.05 were considered significant.

#### Clustering analysis

Bayesian Hierarchical Clustering analysis was performed using the BHC R package version 1.42.0 to separate genes into groups based on their time-dependent expression profile^32^. Input gene expression data into the ‘bhc’ function were 12-h, 36-h and 72-h, versus respective time matched controls (absolute log fold change ≥ 0.66 and adjusted *p* value ≤ 0.05), normalized log ratios and the parameter ‘dataType’ set to ‘time-course’. Optimal clusters were extracted and the quantile distribution of gene sets belonging to each cluster subsequently plotted for visualization of gene expression patterns.

#### Deconvolution analysis

Hippocampus specific single cell transcriptome counts, metadata and tSNE coordinates were downloaded from the UCSC Cell Browser on 10 October 2021: https://cells.ucsc.edu/mouse-nervous-system/hippocampus/. “CA1” specific cells were extracted from the metaData and log normalized, scaled and centered counts using the ‘NormalizeData’ and ‘ScaleData’ (using all genes) functions of Sureat v4.0.3^33^. Uniform Manifold Approximation and Projection (UMAP) dimensionality reduction was performed using the RunUMAP function from Seurat, with arguments ‘reduction’ set to ‘pca’ and ‘dims’ as 1:20, where the input data was the Principle Component Analysis (PCA) performed using the RunPCA function. Clustering analysis of cells was performed using the “FindNeighbours” (input reduction set to ‘pca’, dimensions upto 20) and “FindClusters” (screening resolution of 0.1–1) functions of Sureat. Clustering resolution 0.2 was selected by comparing annotated clusters defined in Zeisel with clustering results, splitting neurons into 4 classes of neurons and 8 clusters in total. Marker analysis was also conducted using the ‘FindMarkers’ function of Sureat for each cluster, with parameters ‘logfc.threshold = 0’ and ‘min.pct = 0’.

Deconvolution of bulk RNAseq data to infer single-cell (SC) population state (or cell abundances) was then performed using the CPM (Cellulation Population Mapping) algorithm implemented in scBio v0.1.6^34^. Inputs to the CPM function of scBio were our RNAseq data described above (normalized counts for each timepoint; treatment and control) with defaults settings. ‘cellSpace’, was the CA1 specific UMAP coordinates generated above; ‘SCLabels’, were which the 8 clusters identified by clustering analysis and ‘calculateCI’ as well as ‘quantifyTypes’ were set to ‘TRUE’. Input data were limited to the genes that were present in both RNAseq and scRNAseq datasets. Predicted alignments of the BulkRNAseq data to cell types was extracted and plot onto CA1 specific UMAP space for each timepoint.

#### Ingenuity pathway analysis

Differentially expressed genes (absolute log fold change ≥ 0.66 and adjusted *p* value ≤ 0.05) with predicted alignments of the BulkRNAseq data to cell types (“Neurons1”, “Neurons2”, “Neurons3”, “Neurons4”, “Oligo”, “Astrocytes”, “Immune” and “Vascular”) were analyzed separately with the use of QIAGEN IPA (QIAGEN Inc., https://digitalinsights.qiagen.com/IPA)^35^. *p* Values of 0.05 were considered significant.

### Ethics approval and consent to participate

All animal procedures were in accordance with the guidelines of the University Hospital Bonn, Animal-Care-Committee as well as the guidelines approved by the European Directive (2010/63/EU) on the protection of animals used for experimental purposes and ARRIVE guidelines.

## Results

### Dynamic mRNA signatures in the early phase of epileptogenesis after Pilocarpine-induced SE

**Figure 1:**
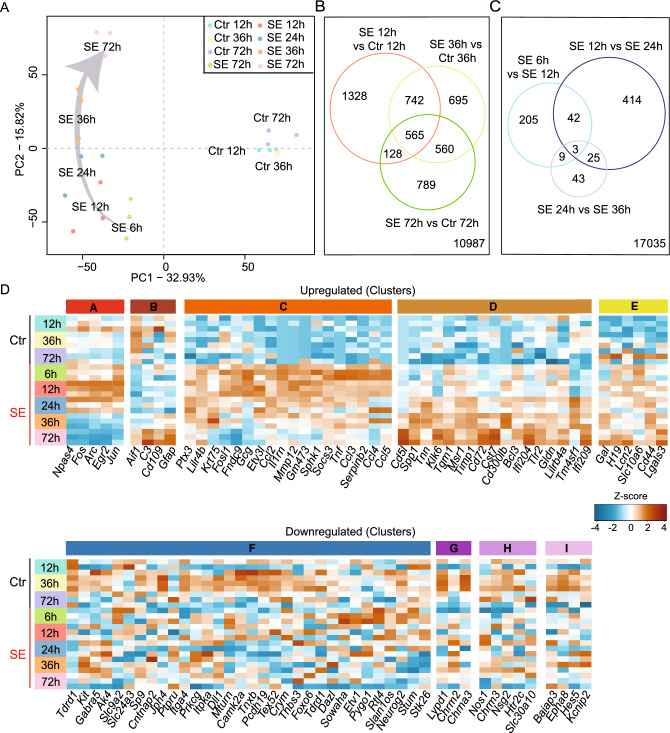
Gene expression profiles after pilocarpine-induced SE follow a time-dependent pattern. (**A**) Principal component analysis (PCA) of control (Ctr) and pilocarpine-induced status epilepticus (SE) samples for each time point (Ctr: 12 h, 36 h and 72 h; SE: 6 h, 12 h, 24 h, 36 h, and 72 h; n = 3 each). (**B**,**C**) Venn diagrams representing the number of differentially expressed genes between (**B**) SE and control samples (*p* ≤ 0.05; Log2FC ≥ |0.66|), and (**C**) all SE samples (*p* ≤ 0.05; Log2FC ≥ |0.66|). (**D**) Heat map of the 100 most differently expressed genes categorized according to their mRNA increase (upregulated clusters **A**–**E**; upper panel) or decrease (downregulated clusters **F**–**I**; lower panel) analysed by k-means clustering.

To decipher transcriptional changes early after pilocarpine-induced SE in the hippocampal CA1 subfield, we compared mRNA expression profiles of pilocarpine-induced SE animals and non-SE controls in hippocampal CA1 at five different time points, i.e. 6, 12, 24, 36 and 72 h after SE. Only minimal transcriptional changes were observed between the non-SE control samples at 12, 36 and 72 h as evidenced by the Principal Component Analysis (PCA) that showed strongly clustered control samples without large differences between the various time points and no overlap with the samples from the SE-experienced mice (Fig. 1A). In contrast, for the SE samples, each time point was strongly clustered, and sequential variability in expression between different time points was observed in the second principal component (PC2), demonstrating a stepwise transcriptomic change along the time axis between 6 and 72 h after SE (Fig. 1A).

Next, volcano plots were generated to illustrate the transcriptomic changes during the early time points after SE. To this end, we analysed the three time points with respective control samples (i.e. 12, 36 and 72 h), allowing a direct comparison between SE and non-SE controls. Of the 18,153 genes analysed, 4680 (12 h), 3640 (36 h), and 2384 (72 h) genes were differentially expressed after pilocarpine-induced SE (*p* < 0.01; Fig. S1A). A deeper analysis of the most prominent changes between the different time points after SE revealed a relatively large number of differentially expressed genes (DEGs) at all three time points (a total of 565 DEGs; upregulated = 468, downregulated = 89, and up- or down-regulated = 8; Fig. 1B, Suppl. Table 1). The highest number of DEGs altered at a specific time point was observed 12 h after SE (1328 DEGs), suggesting rapid expression changes shortly after pilocarpine-induced SE (Fig. 1B). Time point specific expression changes were also observed 36 and 72 h after pilocarpine-induced SE (695 and 789 DEGs respectively; Fig. 1B). In addition to these unique, time-dependent expression differences, common transcriptomic changes were also observed between the different time points (742 DEGs at both 12 and 36 h; 560 DEGs at 36 and 72 h; 1286 DEGs at 12 and 72 h), indicating the presence of a relatively large group of genes that contribute to the process of epileptogenesis over a longer period of time.

Next, we compared the most prominent expression changes between the different time points after SE (Log2FC > I1I; FDR 0.05). All five time points after SE were analysed. Most changes were observed when comparing 12 and 24 h after SE (414 DEGs), followed by comparing 6 and 12 h after SE (205 DEGs) (Fig. 1C, Fig. S1B, Suppl. Table 2). Interestingly, three genes (i.e. *Hlf, Tmem100* and *Cmtm5*) were differentially expressed at all three comparisons (Suppl. Table 2).

### Early epileptogenesis is associated with the up-regulation of immune signalling cascades and transcription factors.

To gain further insight into the major time-dependent changes early after pilocarpine-induced SE in the hippocampal CA1 subfield, we selected the top 100 DEGs between SE and non-SE controls, dividing the group into the 50 most augmented (“upregulated”) and the 50 most decreased (“downregulated”) genes. Using k-means clustering, we grouped these genes based on their expression patterns. For the up- and the down-regulated group, we found five clusters and four main clusters, respectively (Fig. 1D upper panel; clusters A–E up-regulated genes, lower panel; clusters F–I down-regulated genes). STRING analysis of the different clusters revealed that for the upregulated groups, GO terms corresponded to TFs (cluster A) as well as inflammatory processes (clusters B–D). For the downregulated clusters, GO terms were related to the regulation of membrane excitability (clusters F–I) and neurodegeneration (clusters F,I) (Fig. S1C).

**Figure 2:**
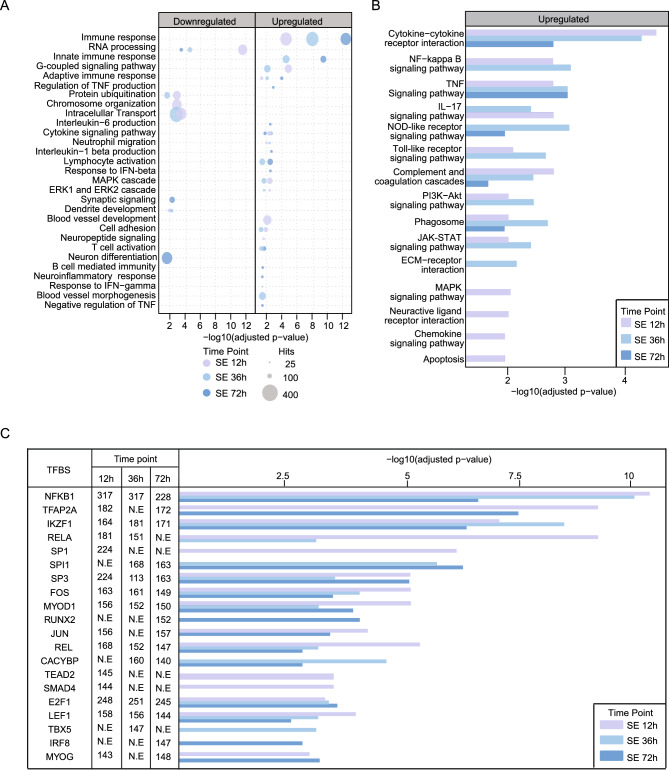
Complex gene expression dynamics in the early phase of epileptogenesis. (**A**) GO analysis of differentially expressed genes (DEGs) (*p* ≤ 0.05; Log2FC ≥ |0.66|) at 12 h, 36 h and 72 h after pilocarpine-induced SE. (**B**) KEGG pathway analysis of DEGs (*p* ≤ 0.05; Log2FC ≥ |0.66|) at 12 h, 36 h and 72 h after pilocarpine-induced SE. (**C**) Transcription factor enrichment from JASPAR and TRANSFAC database (*p* < 0.05) of upregulated DEGs (*p* ≤ 0.05; Log2FC ≥ |0.66|) at 12 h, 36 h and 72 h after pilocarpine-induced SE.

To further unravel the molecular cascades in more detail, additional GO functional annotation analyses were performed on all DEGs at 12, 36 and 72 h after SE. Downregulated DEGs corresponded to pathways associated with GO terms related to chromosome organization, RNA processing, intracellular protein transport, protein ubiquitination, synaptic signalling and neuronal differentiation, indicating a clear reduction in neuronal activity in the early phases of epileptogenesis (Fig. 2A). In contrast, the upregulated genes were mainly enriched in processes of the immune system (Fig. 2A). Genes related to blood vessel development and morphogenesis were strongly activated at 12 h after SE, whereas activation of several specific immunological cascades including interferon beta (IFN-β), interferon gamma (IFN-γ) interleukin-1-beta (IL-1β), interleukin-6 (IL-6) and B cell mediated immunity occurred at the later time points. Interestingly, genes related to the mitogen-activated protein kinases (MAPK) cascade and extracellular signal-regulated kinases (ERK1 and ERK2) showed a strong increase after 12 and 36 h, which was no longer evident at 72 h (Fig. 2A). Heat maps plotting the Log2FC of individual genes confirmed the gradual increase in gene expression for components of the inflammatory IL-1β and IL-6 pathways at all time points, and a temporary increase only at 12 and 36 h for components of the ERK1, ERK2 and MAPK pathways (Fig. S2A).

Subsequent Kyoto Encyclopedia of Genes and Genomes (KEGG) pathway analysis of the upregulated genes confirmed that most KEGG terms were related to inflammatory processes (Fig. 2B). The cytokine–cytokine receptor interaction showed the strongest increase at all three time points (Fig. 2B), indicating that cytokine activation is the main process for cell infiltration and neuroinflammation.

### Transcription factor expression early after pilocarpine-induced SE

Activation of TFs is one of the earliest responses following a brain insult^36^. Recently, we have identified several key transcriptional control mechanisms of CA1 pyramidal burst firing activity, namely transcriptional activation by the transcription factors Metal Regulatory Transcription Factor 1 (Mtf1) and Early Growth Response 1 (Egr1)^7,37^. Consistent with our previous data^8,37^, we observed a significant increase in Mtf1 and Egr1 expression at 12 h after SE, which had returned to basal levels at 24 h (Fig. S2B).

As a first stop to obtain a broader view of the transcriptional regulatory mechanisms associated with aberrant hippocampal intrinsic plasticity on a genome-wide level, a TF enrichment analysis (TFEA) was performed. TFEA prioritizes TFs based on the overlap between the DEGs and annotated TF targets^31^. Comparison of the different time points after SE revealed that most TFs were enriched at all three time points (12, 36 and 72 h after SE), although the strongest overall enrichment was observed at 12 h after SE (Fig. 2C). The highest enrichment score was observed for Nuclear Factor Kappa B Subunit 1 (*Nfkb1*). Two TFs that form heterodimers with Nfkb1, i.e. REL Proto-Oncogene (*Rel*) and RELA Proto-Oncogene (*Rela*)^38^, were also significantly enriched in the early phases after pilocarpine-induced SE. A strong enrichment was also observed for the proto-oncogenes FBJ osteosarcoma oncogene (*Fos*) and Jun (*Jun*), two TFs known as immediate early genes (IEGs), a group of TFs that also includes the previously identified epilepsy-associated TF Egr1 (Figs. 2C, S2B)^7,8^. Interestingly, two TFs critical for microglial activation, i.e. Runt-related transcription factor 2 (*Runx2*) and interferon regulatory factor 8 (*Irf8*)^39,40^, were enriched only after 72 h, suggesting that microglial activation occurs at this later time point. Intriguingly, a more detailed analysis of the microglial and astrocytic markers confirmed the markedly increased expression at this later time point (Fig. S2B).

### Early epileptogenesis is defined by highly dynamic DEG clusters associated with distinct immune responses and hyperexcitability

**Figure 3:**
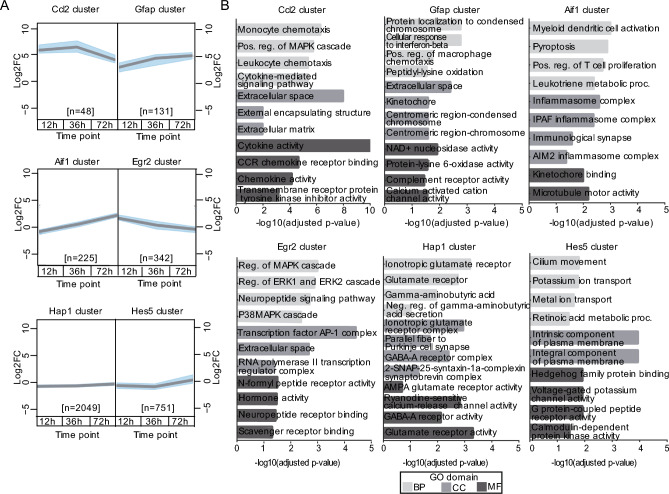
Highly dynamic gene expression clusters associate with a time-dependent immune response. (**A**) Bayesian Hierarchical Clustering analysis of DEGs (*p* ≤ 0.05; Log2FC ≥|0.66|) identified distinctive time-dependent gene activation patterns. Clusters aligned to k-means clustering were selected for further analysis. Grey lines represent the median, and blue shading the upper and lower quantiles. (**B**) GO analysis (Biological Process, Cellular Components and Molecular Functions) for DEGs in each of the six clusters depicted in (**A**).

To further evaluate similar changes in mRNA levels in the time series during early epileptogenesis, we then applied a model-based Bayesian hierarchical clustering (BHC) algorithm to the time series. In total, 30 gene clusters were found by grouping DEGs (FDR ≤ 0.05; Log2FC ≥ |0.66|, Fig. S2C, Suppl. Table 3). Most of these clusters showed a rather stable expression pattern or presented only a minimal change over time. However, a few clusters, revealed a more remarkable expression pattern in time (Fig. S2C). We selected the most interesting BHC clusters, i.e. clusters 1, 7, 16, 23, 28 and 30 (Fig. 3A), and investigated the most relevant GO terms for these clusters.

“Cluster 1” showed a strong increase in gene expression in SE animals compared to the corresponding controls at all time points, with the strongest increase at 12 and 36 h after SE. This cluster also contained the genes originally found in cluster “C” (Fig. 1D) and was named after the gene with the highest Log2FC within this group, i.e. the “Ccl2 cluster” (Fig. 3A). Consistent with the results described above, cytokine activity, monocyte chemotaxis and activation of the MAPK cascade were found to be highly relevant for the genes found within this cluster (Fig. 3B, “Ccl2 cluster”). “Cluster 7”, containing the genes *Gfap, CD109* and *C3*, was termed the “Gfap cluster” and contained genes that showed a gradual increase in gene expression over time (Fig. 3A). Within this cluster, GO terms such as complement receptor activity and response to interferon-beta were observed (Fig. 3B, “Gfap cluster”), confirming the gradual increase through time for inflammation-related processes in time. “Cluster 16” was named after the key microglial marker allograft inflammatory factor 1 (*Aif1;* “Aif1 cluster”) and contained genes that showed the largest differences at 72 h after SE (Fig. 3A). As with the “Gfap cluster”, GO terms related to inflammatory processes, including the inflammasome complex and T cell proliferation, were also evident in this cluster (Fig. 3B, “Aif cluster”). “Cluster 23”, named after the gene with the highest Log2FC at 12 h, i.e. Egr2, contained mostly TFs and showed the strongest increase in gene expression at 12 h (Fig. 3A, “Egr2 cluster”). In addition to GO terms related to the transcriptional machinery, GO terms related to the MAPK, ERK1 and ERK2 signalling cascades were also observed for this cluster (Fig. 3B, “Egr2 cluster”).

“Clusters 28 and 30” contained most of the genes belonging to the “downregulated” group originally found in clusters F–I (Fig. 1D). These two clusters were named after the most strongly downregulated genes within them: Huntingtin Associated Protein 1 *(Hap1)* for “cluster 28” (“Hap1 cluster”) and *Hes5* for “cluster 30” (“Hes5 cluster”). For the “Hap1 cluster”, most of the observed GO terms belonged to GABA and glutamate receptor signalling and for the “Hes5 cluster”, cascades related to potassium and metal ion transport were found (Fig. 3B).

### Identification of specific transcription factor sets orchestrating immune versus ion channel signalling

**Figure 4:**
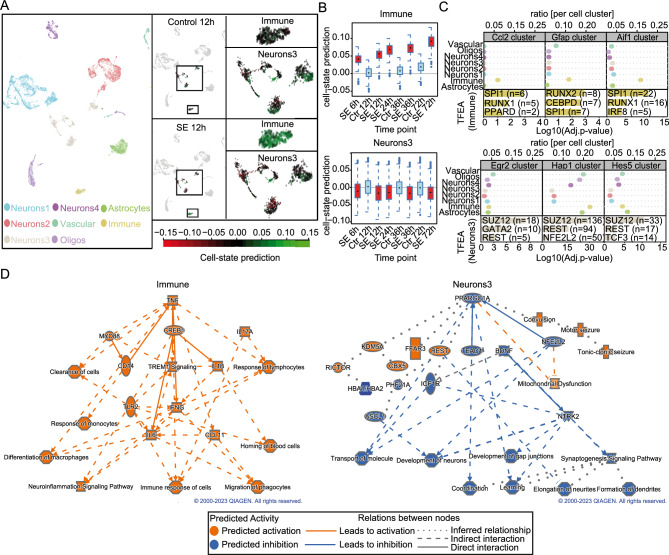
Distinct cell populations are associated with upstream regulators triggering immune response and neuronal dysfunction. (**A**) Uniform manifold approximation and projection (UMAP) of single cell data from CA1 hippocampal cells with eight defined clusters (left panel). Representative UMAP example of SE 12 h reveals distinct cell states compared to control 12 h, mainly originating from the “immune” and “neurons3” clusters (right panels). (**B**) Cell-state prediction of the “immune” (upper panel) and “neurons3” (lower panel) clusters of control (12 h, 36 h and 72 h) and SE (6 h, 12 h, 24 h, 36 h and 72 h). (**C**) Proportion of individual cell clusters belonging to six selected BHC clusters. TFEA depicts predictive TFs regulators for each cluster for “Immune” population (upper panel) and “Neurons3” (lower panel). n-numbers indicate the numbers of genes associated with each TF. (**D**) Graphical summary of the IPA analysis of the “Immune” and Neurons3″ populations. The graphical summaries were generated using the QIAGEN IPA software v01-22-01 (QIAGEN Inc., https://digitalinsights.qiagen.com/IPA).

To gain a deeper understanding of the major cell types and cascades involved in the hippocampal CA1 subfield during epileptogenesis, a deconvolution analysis was performed based on the CA1 cell dataset of Zeisel et al.^41^. Four neuronal populations with distinct transcriptomic patterns in time were isolated (“Neurons1–4”; Fig. S3A). Also, astrocytic (“Astrocytes”), oligodendrocytic (“Oligos”), vascular, and immune populations were found (Figs. 4A; S3A). The “Immune”, “Oligos” and “Vascular” populations showed a higher cell state prediction index after SE, suggesting an augmentation in cell type specific genes, whereas the “Astrocytes”, “Neurons3” and “Neurons4” populations showed a diminution at the mRNA level (Fig. S3A). Interestingly, the “Neurons3” and “Immune” populations had the largest variation in the cell status prediction index (Figs. 4B, S3B), indicating that these specific cell types may play an important role in the early phases of epileptogenesis.

Next, in order to correlate the highest variations in specific cell types (“Neurons3” and “immune”) with mRNA temporal dependency, the resulting cell type populations were combined with our BHC algorithm to determine the individual contribution of each cell-type to the different temporal clusters, (Fig. S4A). Among our six selected clusters (Fig. 3A), the immune-related clusters (the “Ccl2”, “Gfap” and “Aif” clusters; Fig. 3B) were largely driven by an increased ratio of “immune” cell types (Fig. 4C; upper panel). In contrast, the “Egr2”, “Hap1” and “Hes5 clusters” possessed a mixture of cell type populations (Fig. 4C, lower panel). To identify TFs that regulate these subgroups of genes, additional TFEA analysis was performed. By grouping genes with similar expression changes over time, genes that are likely to be co-regulated by the same TFs can be identified^42^. Interestingly, the “Immune” population is strongly influenced by specific TFs that regulate individual clusters with distinct time-dependent patterns. Indeed, the transcription factor PU.1 (*Spi1*) was found to be enriched in all three immune-related clusters. In addition, two members of the Runt-related transcription factor family (i.e. *Runx1* and *Runx2*), were also highly enriched in all three clusters (Fig. 4C; left panel). On the other hand, the “Egr2”, “Hap1” and “Hes5” clusters showed a strong association with the Suppressor of Zeste 12 Protein Homolog (*Suz12*) and Neuron Restrictive Silencer Factor (*Rest*) TFs (Fig. 4C; right panel).

As a next step, we used Ingenuity Pathway Analysis (IPA) Upstream Regulator Analysis to identify the upstream molecules that may be responsible for gene expression changes observed in each of our populations. In the case of the “non-neuronal” populations, regulators like tumour necrosis factor (*Tnf)*, transforming growth factor-beta (*Tgf-β1),* cAMP responsive element binding protein 1 (*Creb1*), *IL-1β, Ifn-γ, and IL-6* highly overlap with the DEGs of all these cell populations (Fig. S4B; Left panels). In fact, these regulators are presented among the most significant entities in the graphical IPA summary of the "Immune" population (Fig. 4D). Here, strong interactions between these molecules and their predicted roles in the activation of major biological themes as “Neuroinflammation signalling pathway” and “Differentiation of macrophages” are evident. Conversely, in the “neurons3” population, predicted inhibition on the “Development of neurons” and “transport of molecules” seems to be orchestrated by the activation of *Rest* and the inhibition of Nuclear Factor Erythroid 2-Related Factor 2 (*Nfe2l2)* and Peroxisome Proliferator-Activated Receptor Gamma Coactivator 1-Alpha (*Ppargc1a)* (Fig. 4D). This is further reflected in the Upstream Regulator Analysis of other neuronal populations with high overlap of *Rest* and *Nfe2l2* (“neurons1” and “neurons4”) (Fig S4B; Right panels). Interestingly, we observe overlap of *Creb1* with the genes within several populations (“Oligo”, “Astrocytes, “immune”, “Neurons1”, “Neurons2” and Neurons4”) in a predicted activated state (Fig. S4B). These analyses have uncovered novel immune-related epileptogenesis-associated TFs, as well as TFs involved in the regulation of channelopathies in the hippocampal CA1 region early after pilocarpine-induced SE.

## Discussion

Despite its transient nature, SE represents a fundamental insult to the affected brain structures. SE is immediately detrimental and induces long-term neuronal network reorganization that predisposes to chronic seizures. An in-depth understanding of the key transcriptional signalling cascades directly triggered by SE may therefore provide new insights into the pathomechanisms that initiate epileptogenic processes.

**Figure 5:**
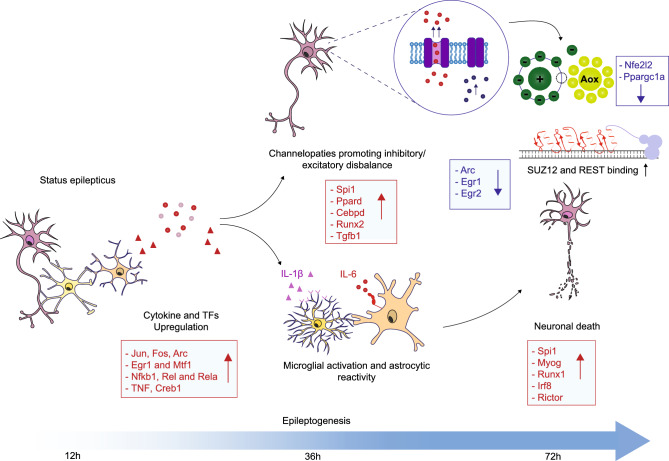
Status epilepticus promotes epileptogenesis and is associated with altered expression of distinct transcription factors. Main Transcription factors (TFs) altered during the epileptogenic process. These TFs are involved in inflammatory signalling cascades mediated by microglial activation, reactive astrocytes and ion channel signalling.

While the functional annotation of the downregulated genes early after SE in CA1 (12 h) suggests alterations in non-cell-type-specific processes such as “chromosome organization”, “intracellular transport”, and “protein ubiquitination”, two major sets of transcript clusters represent substrates of induced (a) innate inflammation and (b) complex shifts in neuronal excitation. Both clusters appear to be regulated by distinct sets of only a few TFs. Our overall non-cell-type-specific TF analysis revealed a rather limited number of relevant TFs, including components of the Nfkb pathway (e.g. *Nfkb1*, *Rel* and *Rela*) and several IEGs (e.g. *Jun, Fos, Egr1, Egr2, Npas4* and *Arc*), to play a key role in organizing gene expression modules at the earliest stages post-SE (Fig. 5).

### Inflammatory clusters

Previous transcriptomic studies on post-SE models have reported alterations mainly related to immune and inflammatory responses^3,43–45^. In fact, the involvement of the MAPK and ERK signaling pathways in the epileptogenic process early after SE has been reported previously^45,46^. These findings are in line with our results, as we noted a gradual decrease in time on the activation of these signalling pathways. In contrast, two other main processes, i.e. “interleukin 1β production” and “interleukin 6 production”, both involved in the activation of the microglial-astrocyte axis in epileptogenesis^47^ were augmented during the early phase after SE. Furthermore, also expression of the key microglial markers *Aif-1* and *Tmem-119*^48^ was gradually increased over time.

To date, it is still controversial whether activation of microglia after an acute stimulus, such as pilocarpine-induced SE, has neuroprotective effects^49–53^ or contributes to neuroinflammation^54–59^. Previous studies, describe the role of Ifn-γ, Tnf, IL-1β and IL-6 as master regulators of microglia immunomodulation in early epileptogenesis^60,61^, and as known activators of the “M1” polarization state^62^. M1 polarization is mainly associated with pro-inflammatory effects, whereas M2 activation seems to be related to neuroprotective cascades of microglia^63,64^. Our IPA analysis showed association with the TREM1 signalling pathway; a key pathway in inflammatory processes^65,66^ and known to enhance neuroinflammation by modulating microglia polarization^65^. The potent M1 polarization at these early stages after SE suggests a predominant pro-inflammatory microenvironment predisposing the progression of epileptogenesis. Intriguingly, our denoted “Gfap” and “Aif1” clusters showed enrichment of several innate immune processes, including “pyropoptotic processes”, “inflammasome complexes”, and “complement receptor activity”. These processes are also related to M1 microglial polarization and astrocytic reactivity^67,68^. These findings are in line with other studies suggesting that initially activated microglia can be responsible for the induction of epileptogenic reactive astrocytes^54,69^. In addition, a recent study showed that the complement C3-C3aR pathway mediates interaction between microglia and astrocytes after SE^70^. In our study, C3 and C3aR are also strongly upregulated mainly 36 and 72 h after SE and cluster together within the “Gfap” cluster, suggesting an astrocytic activation derived from the C3-C3aR pathway in the pilocarpine-SE model.

These results imply that the time-dependent activation and inactivation of genes that enrich immunological cascades in the early phases of epileptogenesis stimulate processes such as microglial activation. Moreover, our study highlights the role of microglia and particularly, the key role of the astrocytic-microglial axis in epileptogenesis and its transition into a pro-inflammatory phenotype.

### TFs controlling inflammatory clusters

Our TF analysis after deconvolution and IPA analysis revealed that the TFs *Spi1, Irf8, Cebpb* (CCAAT/enhancer binding protein beta)*, Ppard* (peroxisome proliferator activator receptor delta)*,* and two members of the *Runx* family (i.e. *Runx1* and *Runx2*) correlated strongly with alterations in immune processes. *Spi1* is critical for microglial survival, but also plays a key role in the development of macrophages, neutrophils, and T and B cells^71,72^. In addition, it has been reported to work in concert with *Irf8* to activate microglia^73^. These data suggest that a very small set of TFs induces distinct inflammatory cascades and is associated with the activation of mainly reactive glia-mediated immune mechanisms shortly after SE. At the cellular level, innate inflammatory astroglia and microglia, rather than adaptive immune cells constitute the major early cellular infiltrates in CA1 affected by an epileptogenic brain insult. Clearly, microglial and astroglial infiltrates created a persistent immune stress environment in the affected CA1, and as the disease progresses further, induced neuronal plasticity becomes more important; the transcriptional profiles recapitulate the transition from early, more general metabolic and immune cellular processes to altered neuronal mRNA signatures, showing the switch from the initial insult, affecting non-cell type-specific processes to direct epileptogenic effector alterations of individual neuronal populations. The integration of these processes and transcription factors point to the hypothesis that a limited number of upstream regulators, for example Creb1, might orchestrate the transcriptomic alterations of neuronal and non-neuronal populations.

### Excitability clusters

This interpretation is based on the finding that 72 h after SE, primarily neuron-associated gene sets (including “neuronal differentiation” and “synaptic signalling”) were observed to be altered. The most strongly affected neuronal populations are clustered within the “Hap1” and “Hes5” clusters. Alterations were found at multiple levels, including changes in potassium ion transport, G protein-coupled peptide receptor activity, hedgehog signalling, and calmodulin-dependent protein kinase activity, confirming the contribution of these cascades in the process of epileptogenesis^74–78^. Moreover, the functional annotation of the “Hap1” cluster to GABA and glutamate receptors may imply a more general imbalance in inhibitory and excitatory synaptic transmission. These results imply that changes at the transcriptional level promote mechanisms that dysregulate genes associated with excitability, and thus promote channelopathies even in this early stage of epileptogenesis. These early changes in ion channel gene expression are also visible in the single-gene expression data for several ion channels; including the ion channels Gabra5, Chrna3, Chrm2/m3 and Kcnip2.

### TFs controlling excitability clusters

Augmentation of IEG expression strongly correlates with neuronal activity^79^. After an initial prominent increase in expression of the IEGs promoting aberrant ion channel gene expression, most IEGs return back to basal expression levels within 72 h. The transcriptional repressors *Suz12* and *Rest* were related to the genes showing a downregulation early after pilocarpine-induced SE and were strongly associated with hyperexcitability and GABA/glutamate receptor activity. Both TFs are master regulators of gene silencing, and both have been reported to be differentially expressed in the early phases of epileptogenesis in other animal models^80,81^. Our results revealed a strong augmentation of *Rest* mRNA levels mainly 12 h after SE. For *Suz12*, less variation in mRNA expression was detected early after pilocarpine-induced SE. However, prolonged TF enrichment of the downregulated genes associated with *Suz12* was observed, implying a complex interaction between *Suz12* and its target genes. These findings reveal a complex interaction between different pathways and TFs that promote aberrant neuronal development and communication. Moreover, our IPA analysis showed that several neurodegenerative and hyperexcitable processes are predicted to be driven by *Nfel2l2* and *Ppargc1a*. Nfel2l2 is a transcription factor that regulates the expression of antioxidant genes like Ppargc1a^82^. Therefore, these genes are involved in orchestrating the cellular response to oxidative stress^83^. Targeted delivery of antioxidant genes demonstrate the potential to ameliorate other diseases characterized by oxidative stress-induced cell degradation^84^, indicating the potential neuroprotective effects of *Nfel2l2* and *Ppargc1a* in the process of epileptogenesis*.*

Our findings demonstrate vast transcriptomic changes that occur in early epileptogenesis in a complex network of altered transcription factors and pathways. We clearly identify the responsible TFs and TF-dependent transcriptional signatures that mediate the processes at the earliest stages after SE and provide targets for future therapeutically intervention to halt epileptogenesis. Furthermore, our results point to possible interventions on upstream regulators (i.e. *Tnf, Creb1, IL-1β, Ifn-γ, IL-6, Nfel2l2,* and *Rest*) that are involved in multiple pathogenic processes across different cell populations. Nevertheless, we point out to the complexity of simultaneous processes in early epileptogenesis. On the one hand, promoting neuroinflammation, where we have found new possible therapeutic targets associated with the astrocytic-microglia axis including the Nfkb and Trem1 signalling pathway, as well as the multiple cytokines involved in neuroinflammation (Tnf, IL-1β, Ifn-γ and IL-6). On the other hand, transcription factors associated with hyperexcitability (Rest, Gata2 and Suz12) as well as genes associated with the reduction of oxidative stress (Ppargc1a and Nfel2l2).

### Supplementary Information

Supplementary Figure 1.

Supplementary Figure 2.

Supplementary Figure 3.

Supplementary Figure 4.

Supplementary Table 1.

Supplementary Table 2.

Supplementary Table 3.

## Data Availability

The RNA-seq datasets generated and analysed during the current study are available in the Gene Expression Omnibus (GEO) repository, with the GEO accession number GSE241219.

## Abbreviations

BHC: Bayesian hierarchical clustering
DEG: Differentially expressed gene
ERK: Extracellular signal-regulated kinases
GO: Gene ontology
IEG: Immediate early genes
IRF8: Interferon regulatory factor 8
KEGG: Kyoto encyclopedia of genes and genomes
MAPK: Mitogen-activated protein kinases
NFKB1: Nuclear factor kappa B subunit 1
PCA: Principal component analysis
REST: Neuron restrictive silencer factor
RUNX: Runt-related transcription factor
SE: Status epilepticus
SPI1: Transcription factor PU.1
STRING: Search tool for the retrieval of interacting genes/proteins
SUZ12: Suppressor of Zeste 12 protein homolog
TFEA: Transcription factor enrichment analysis
TLE: Temporal lobe epilepsy
