# Supplementary material for: Highly dynamic inflammatory and excitability transcriptional profiles in hippocampal CA1 following status epilepticus
Source: Sci Rep. 2023 Dec 14;13:22187. doi: 10.1038/s41598-023-49310-y (PMC10719343; doi:10.1038/s41598-023-49310-y)
Supplement: Supplementary file 1 — Supplementary Figure 1. [file 41598_2023_49310_MOESM1_ESM.pdf]

Supplementary Figure 1

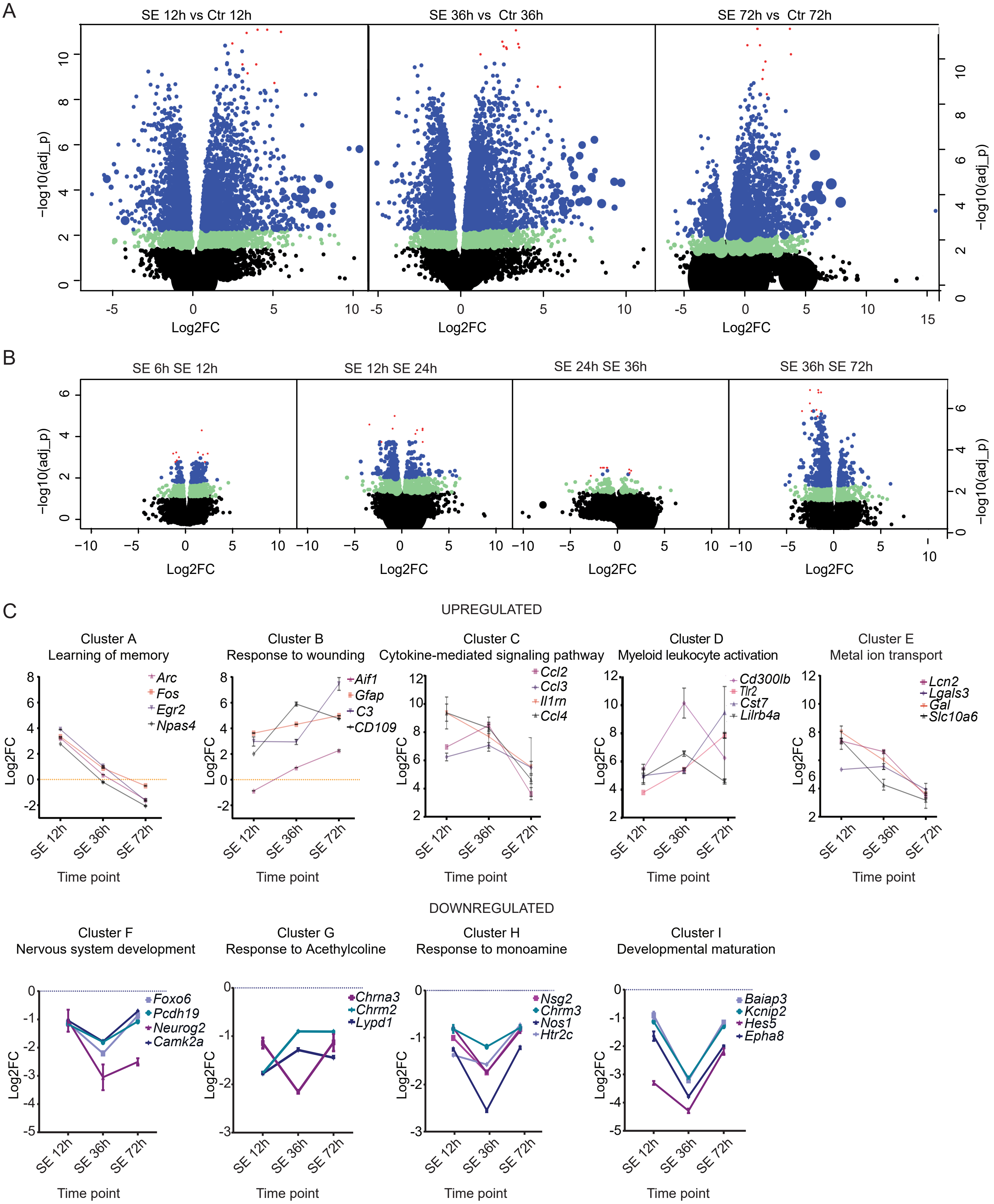

Supplement Fig. 1. Vast time-dependent transcriptomic alterations are strongly influenced by activation/inactivation of distinct signalling pathways. (A, B) Volcano plots illustrate the correlation between statistical significance (Adj. p value) and magnitude of change (Log2FC) of the (A) DEGs comparing SE samples with corresponding controls 12, 36 and 72 hours after pilocarpine-induced SE , and (B) between different time points after SE. (C) Functional protein association networks of the ten different clusters (upregulated clusters A-E; downregulated clusters F-I). STRING analysis depicts the GO terms with the highest score and the four genes with the highest Fold Change of each cluster.
