# Supplementary material for: Highly dynamic inflammatory and excitability transcriptional profiles in hippocampal CA1 following status epilepticus
Source: Sci Rep. 2023 Dec 14;13:22187. doi: 10.1038/s41598-023-49310-y (PMC10719343; doi:10.1038/s41598-023-49310-y)
Supplement: Supplementary file 2 — Supplementary Figure 2. [file 41598_2023_49310_MOESM2_ESM.pdf]

Supplementary Figure 2

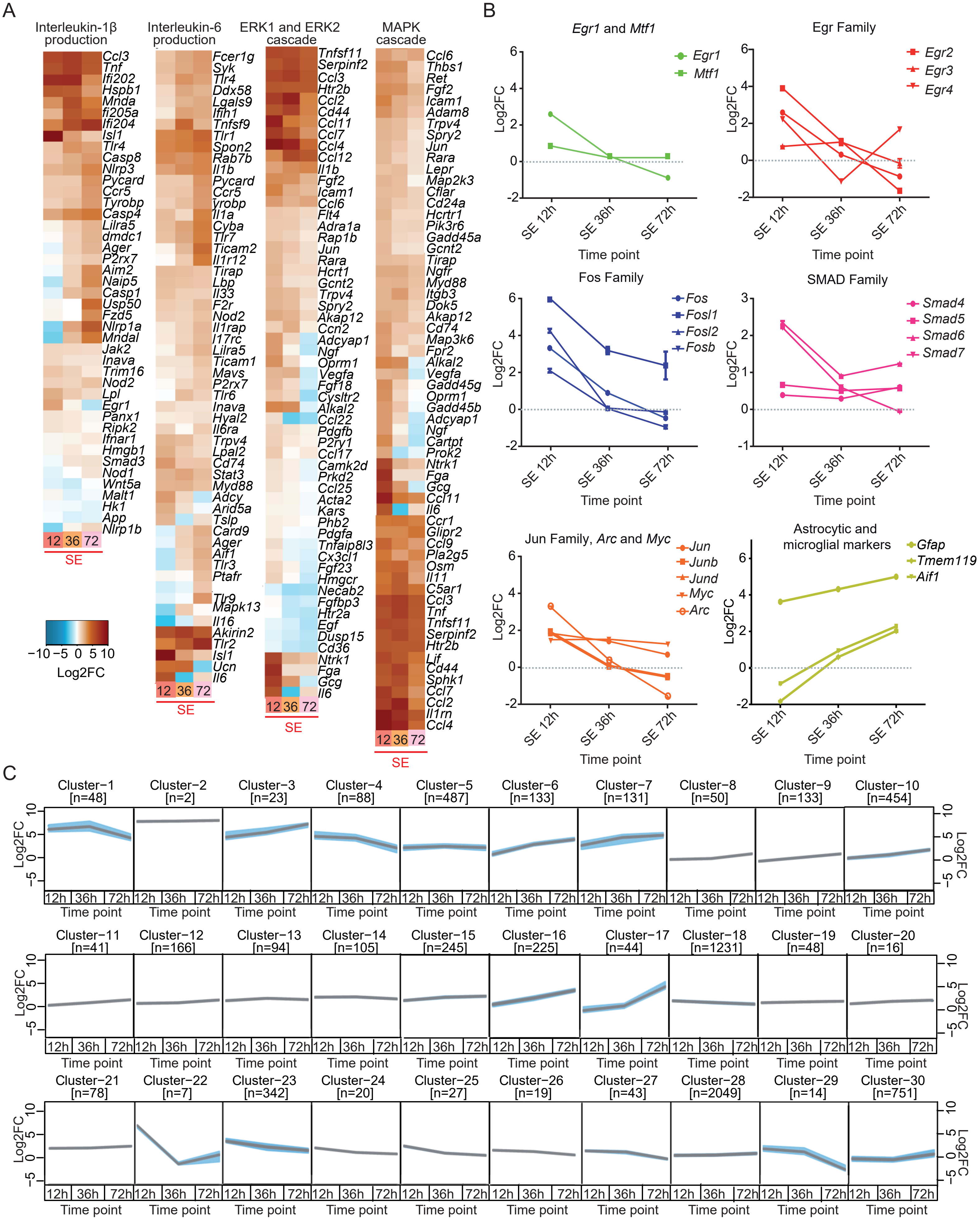

Supplementary Fig. 2. Clustering analysis of differentially expressing genes reveal distinct temporally altered patterns that correlate with altered gene expression of immediate early genes.(A) Heatmaps of gene expression profiles grouped in different cascades (interleukin-1β production, interleukin-6 production, ERK1 and ERK2 cascade, and MAPK cascade). (B) Temporal changes in differentially expressed immediate early genes (IEGs) grouped by transcription factor family (Fos, Egr, SMAD, Jun) and Myc, Arc, Mtf1 as well as astrocytic (GFAP), and microglial activation markers (TMEM-119 and AIF-1). (C) Bayesian Hierarchical Clustering (BHC) analysis of DEGs (p≤0.05; Log2FC≥l0.66l) for each time point. The analysis reveals 30 distinct clusters that exhibit different time-dependent patterns during epileptogenesis
