# Supplementary material for: Highly dynamic inflammatory and excitability transcriptional profiles in hippocampal CA1 following status epilepticus
Source: Sci Rep. 2023 Dec 14;13:22187. doi: 10.1038/s41598-023-49310-y (PMC10719343; doi:10.1038/s41598-023-49310-y)
Supplement: Supplementary file 3 — Supplementary Figure 3. [file 41598_2023_49310_MOESM3_ESM.pdf]

Supplementary Figure 3

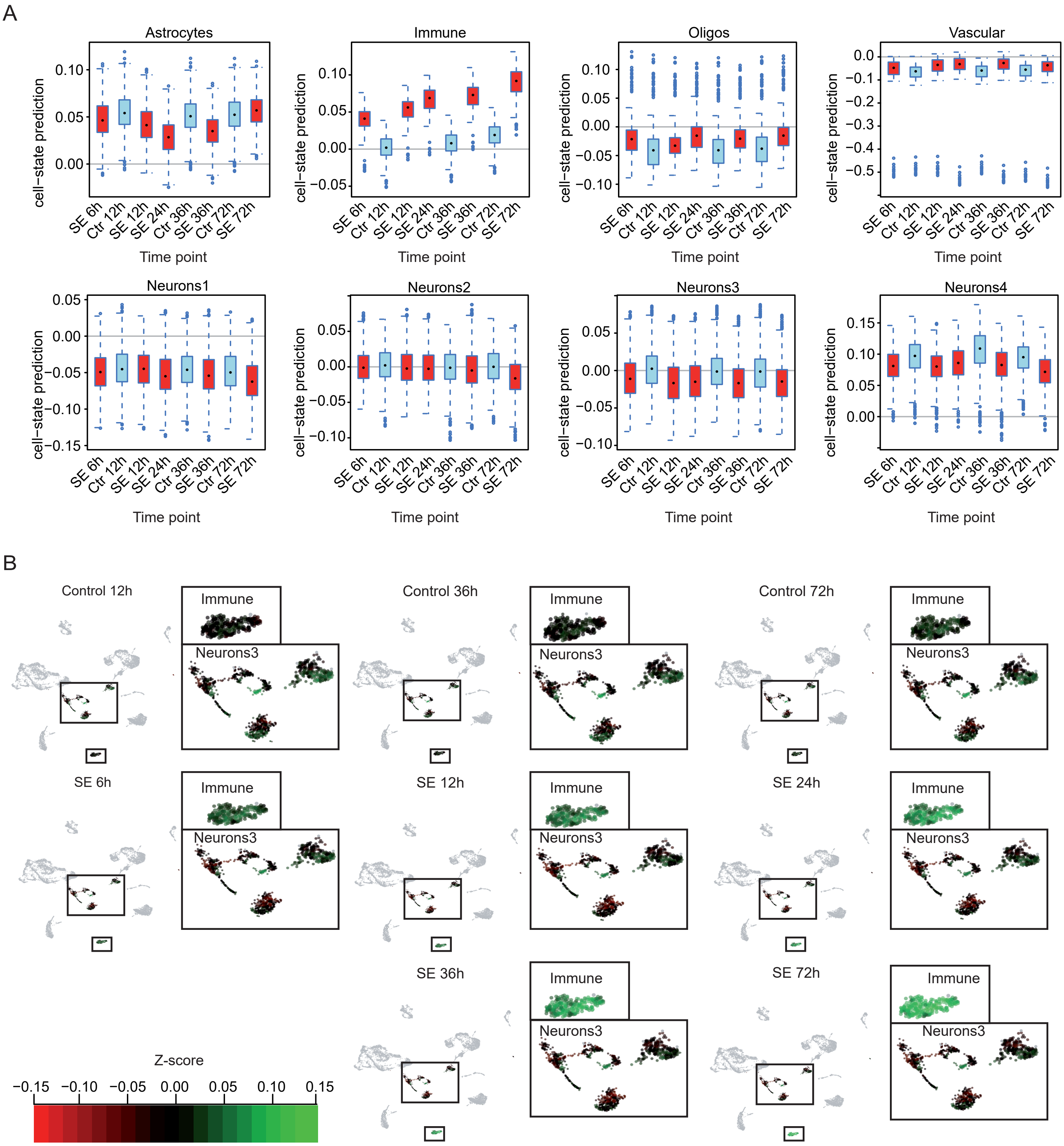

Supplement Fig. 3. Cell-type associated clusters reveal predominant transcriptomic changes associated with the immune cell type population. (A) Bar graphs of cell status prediction show the changes of different cell populations observed after deconvolution analysis. (B) Recomputed Uniform Manifold Approximation and Projection (UMAP) plots for each sample after deconvolution analysis of the CA1 cell dataset. Each point represents a cell cluster assignment provided as a “Class”. Inserts indicate higher magnification of the regions with the highest cell state prediction index after pilocarpine-induced SE.
