# Supplementary material for: Highly dynamic inflammatory and excitability transcriptional profiles in hippocampal CA1 following status epilepticus
Source: Sci Rep. 2023 Dec 14;13:22187. doi: 10.1038/s41598-023-49310-y (PMC10719343; doi:10.1038/s41598-023-49310-y)
Supplement: Supplementary file 4 — Supplementary Figure 4. [file 41598_2023_49310_MOESM4_ESM.pdf]

Supplementary Figure 4

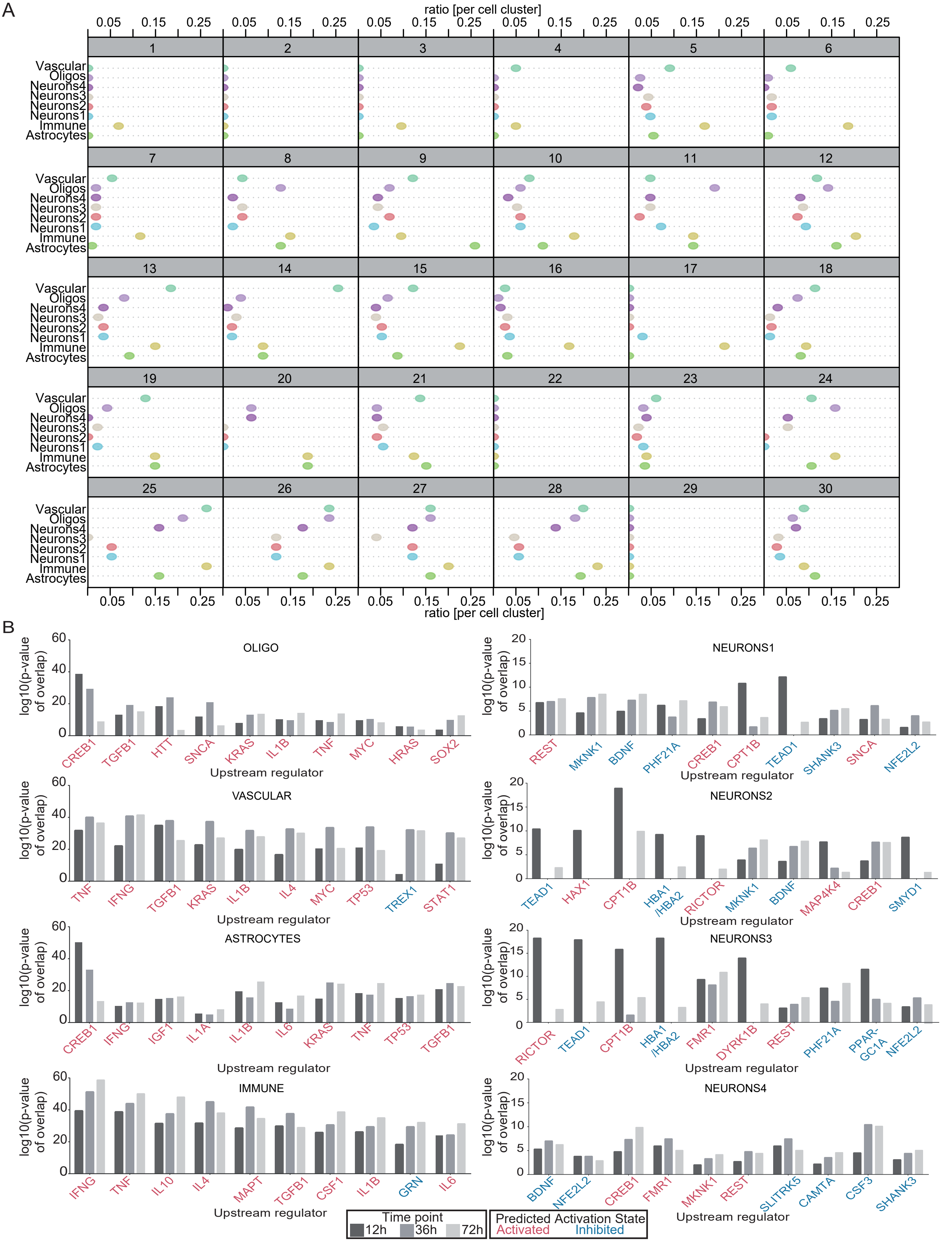

Supplement Fig. 4. Clustering analysis and cell-type associated clusters reveal a predominant immune cell type population associated to specific upstream regulators (A) BHC clustered genes were associated with specific CA1 cell types. The ratio represents the proportion of related cell type genes that are significant markers for each cluster (p<0.05). (B) Comparison of the most relevant upstream regulators for each cell populations at different time points. (p-value of overlap<0.05)
